# Perinatal outcomes following transfer of human blastocysts vitrified at day 5, 6 and 7

**Published:** 2009-10-20

**Authors:** Kenichiro Hiraoka, Kaori Hiraoka, Miyuki Miyazaki, Emi Fukunaga, Toshitaka Horiuchi, Tomoyo Kusuda, Shinichiro Okano, Masayuki Kinutani, Kazuo Kinutani

**Affiliations:** 1 Kinutani Women's Clinic, 8-23-4F, Hondori, Naka-ku, Hiroshima 730-0035, Japan; 2 Graduate School of Applied Biosciences, Hiroshima Prefectural University, 562, Nanatsuka, Shohbara, Hiroshima 727-0023, Japan

## Abstract

Little information is available on perinatal outcome of cryopreserved day-7 blastocyst transfer. In the present report, perinatal outcomes for transfers of cryopreserved blastocysts by a vitrification method were examined with respect to the day of blastocyst expansion among on day 5, 6 or 7 before cryopreservation. We investigated 263 cycles of vitrified-warmed blastocyst stage embryo transfer performed between April 2005 and April 2009, which were reviewed retrospectively. There were 144 cycles with day-5 blastocyst, 100 cycles with day-6 blastocyst, and 19 cycles with day-7 blastocyst transfers. There were no differences among the vitrified day-5, day-6 and day-7 blastocyst transfer groups regarding mean number of embryos transferred, pregnancy rate, implantation rate and miscarriage rate. At this time, 71 deliveries have occurred with no reported abnormalities. There were 47 infants from 41 deliveries with day-5 blastocyst, 26 infants from 23 deliveries with day-6 blastocyst, and 8 infants from 7 deliveries with day-7 blastocyst. There were no differences among the three groups in the mean gestational age, preterm delivery rate and mean birth weight. These results indicated that blastocysts have similar inherent viability regardless of whether they develop by day 5, 6 or 7.

## Introduction

Previous investigators have suggested that the age of blastocyst is indicative of embryo quality among human embryos derived from assisted reproductive technology. Superior pregnancy rates were reported with fresh transfer occurring at day 5 as compared with day 6 [1–3]. However, blastocysts cryopreserved on day 5 have a pregnancy potential similar to those of day-6 blastocysts [4, 5]. These results suggest that differences in pregnancy rate between fresh and cryopreserved day-6 blastocysts attributed to reduced embryo quality with slower development may instead result from impaired endometrial receptivity by the time more slowly growing embryos develop to the stage at which implantation is possible. On the other hand, little published information is available regarding the transfer of day-7 blastocysts because embryos developing slowly are generally considered nonviable and are therefore discarded. Case reports of both fresh and cryopreserved embryo transfers have confirmed that day-7 blastocysts may still be viable [6–8]. However, as far as is known, studies evaluating the relationship between the blastocyst age as late as on day 7 and embryo quality (independent of the timing of endometrial receptivity) are limited to a single report published by Richter *et al*. [9]. These authors evaluated the viability and implantation potential of cryopreserved blastocysts by a slow-freezing method according to the day of blastocyst expansion among on day 5, 6 or 7 before cryopreservation. Additionally, little is available on perinatal outcome of cryopreserved day-7 blastocyst transfer. In the present report, perinatal outcomes for transfers of cryopreserved blastocysts by a vitrification method were examined with respect to the day of blastocyst expansion among on day 5, 6 or 7 before cryopreservation.

## Methods

Cycles of vitrified-warmed blastocyst stage embryo transfer performed between April 2005 and April 2009 were reviewed retrospectively. The treatment was conducted with patients following informed consent. As the study was retrospective, Institutional Review Board approval was not required.

Women were treated with gonadotrophin-releasing hormone (GnRH) analogue buserelin acetate (Mochida, Tokyo, Japan) from either the preceding mid-luteal phase in a long treatment protocol or second day of the cycle in a short treatment protocol. Ovarian stimulation was carried out with human menopausal gonadotrophin (Nikken, Tokyo, Japan) or urinary FSH (Fertinorm; Serono, Japan). Follicular development was monitored with serial (every third day) vaginal ultrasound examinations and serum oestradiol measurements. Human chorionic gonadotrophin (hCG; Teizo, Tokyo, Japan) was administered to women when dominant follicles reached a diameter of 18mm. Oocytes were collected 35h after hCG administration using a vaginal ultrasound-guided procedure and were incubated in human tubal fluid (HTF) medium (Irvine Scientific, CA, USA) containing 10% (v/v) serum substitute supplement (SSS; Irvine). Sperm preparation was carried out using discontinuous ISolate™ (Irvine) gradient. Mature oocytes were either inseminated with spermatozoa 5–7h after oocyte retrieval at a concentration of 100,000 to 200,000 motile spermatozoa/ml for 5 to 10 oocytes or microinjected with a single spermatozoon. The day of oocyte retrieval was defined as day 0. Fertilization was confirmed at 24–25h after oocyte retrieval (day 1) by the presence of two pronuclei. Fertilized oocytes were transferred and cultured in Blast Assist Medium 1 (Medicult, Jyllinge, Denmark) until day 3, and then placed in Blast Assist Medium 2 (Medicult) until day 5. On day 5, the embryos were examined for development into blastocysts and then placed in fresh Blast Assist Medium 2 (Medicult) until day 7. All oocytes and embryos were incubated at 37 °C in an atmosphere of 6% CO_2_, 5% O_2_ and 89% N_2_.

Expanded blastocysts were vitrified on day 5, 6, or 7, depending on day of expansion. For expanded blastocysts, the development of the inner cell mass (ICM) and trophectoderm was assessed. The ICM grading was as follows: A: tightly packed, many cells; B: loosely grouped, several cells; C: very few cells. The trophectoderm grading was as follows: A: many cells forming a tightly knit epithelium; B: few cells; C: very few cells forming a loose epithelium [10]. Only expanded blastocysts scoring B or higher both ICM and trophectoderm grades (*i.e*. BB) were vitrified. The expanded blastocysts were vitrified by the method developed by Kuwayama *et al*. [11] using a cryotop (Kitazato Supply Co., Fujinomiya, Japan), albeit with slight modifications as described previously [5]. The cryotop consisted of a 0.4 mm wide × 20 mm long × 0.1 mm thick polypropylene strip attached to a plastic handle and equipped with a cover straw. As the base medium, modified HTF medium-HEPES (Irvine) plus 20% (v/v) SSS (Irvine) was used. The equilibration solution contained 7.5% (v/v) ethylene glycol (Sigma Chemical Co., MO, USA) and 7.5% (v/v) dimethyl sulphoxide (Kanto Chemical Co., Tokyo, Japan). The vitrification solution was composed of 15% (v/v) ethylene glycol, 15% (v/v) dimethyl sulphoxide and 0.5 mol/l sucrose (Nacalai Tesque, Inc., Kyoto, Japan). Before starting the vitrification procedure, artificial shrinkage of expanded blastocysts was performed in the equilibration solution. First, pipetting of the expanded blastocyst was started immediately after placing the embryo in 1ml of 30°C equilibration solution with a glass pipette slightly smaller in diameter (~140 μm) than the expanded blastocyst. After confirmation of slight shrinkage of the blastocoele, pipetting was performed with a pipette slightly smaller in diameter than the first one (~100–120 μm). This procedure was repeated two to three times until the blastocoele collapsed completely. After blastocoele contraction, the blastocysts were equilibrated in the same equilibration solution for another 2min before exposure to the vitrification solution. The blastocysts were then incubated in 1ml of 30°C vitrification solution and loaded, within 45s onto the tip of the cryotop with ~1 μl of cryoprotectant solution. Then the cryotop was immediately submerged into liquid nitrogen and under the liquid nitrogen the plastic cover was placed over the strip to provide protection during storage.

The warming procedure was done as follows: The protective cover was removed in liquid nitrogen and the end of the polypropylene strip was immersed directly into 1 ml of 37°C 1.0 mol/l sucrose solution for 1 min. The blastocysts were then transferred into 1 ml of 37°C 0.5 mol/l sucrose solution for 3 min and washed twice in the base medium for 5min. As soon as warming of blastocysts had completed, assisted hatching was performed while the warmed blastocysts remained collapsed (within 5min after warming procedure completed) by the method as described previously [12]. Briefly, embryos were placed under mineral oil within a 50μl microdroplet of 37°C Sperm Washing Medium (Irvine) in a Petri dish and positioned on a phase-contrast inverted microscope stage. Total removal of the zona pellucida was performed using a laser (Zilos-tk Laser, Hamilton Thorne Research, Beverly, MA, USA) and mechanical pipetting. Embryos were stabilized with a holding pipette held at 9 o’clock position, and positioned with the laser target located on the outer edge of the zona. The power of laser was 100% and the pulse duration was 500μs. By using this setting, a 10μm hole was formed in the zona by a single laser application. Multiple irradiations along the convex periphery of the zona from outward to inward were used to form a 10μm opening. Similarly, another opening was formed next to the initial one. This procedure was repeated until a large opening was formed in the zona. The size of the opening was 60-90% of the circumference of the zona, estimated from an area of empty perivitelline space. Approximately 2min per embryo was required to complete the procedure. After forming a large opening, the embryo was released from the holding pipette and aspirated out from the opened zona by mechanical pipetting, and the zona was removed. After the assisted hatching procedure was completed, warmed blastocysts were rinsed several times and were cultured in Blast Assist Medium 2 (Medicult) for further culture until transfer. The post-warming survival of blastocysts was observed 1-2h after warming under a microscope, and re-expanded blastocysts were judged to have survived. Warmed blastocyst transfer was performed in hormonal replacement treatment cycles. All women received transdermal oestradiol (Estraderm 1.4-5.8 mg, Kissei, Tokyo, Japan) with gonadotrophin-releasing hormone analogue for the preparation of the endometrium. The administration of progesterone (vaginal 400mg daily) was initiated when endometrial thickness exceeded 10 mm. Embryo transfer was scheduled on day 5 after the initiation of progesterone treatment. The time from warming to transfer ranged from 2-3h. One to two surviving blastocysts were transferred into the patient’s uterus.

Analysis was restricted to cycles in which all transferred embryos were vitrified on the same day of development, and in which transfers were performed on the same day on which embryos were warmed. For patients undergoing multiple vitrified-warmed blastocyst transfer cycles during the study period, only the first cycle was included in the analysis. A clinical pregnancy was defined as a pregnancy when a gestational sac was recognized in the uterus by ultrasound. The implantation rate was determined by dividing the number of gestational sacs by the number of embryos transferred.

The one-way ANOVA test, chi-squared test and Fisher’s exact test were used as appropriate to determine statistical differences among groups. A P-value of <0.05 was considered significant.

## Results

Data were available for 263 cycles of vitrified-warmed blastocyst transfer that fit the inclusion criteria. There were 144 cycles with day-5 blastocyst, 100 cycles with day-6 blastocyst, and 19 cycles with day-7 blastocyst transfer. One to two blastocysts were warmed and transferred per cycle. Clinical pregnancies resulted from 140 (53.2%) of these treatment cycles.

Table 1 compares cycle characteristics according to the day (after oocyte retrieval) of blastocyst vitrification. There were no differences among the three groups in the age of women at vitrification or warming, survival rate, mean number of embryos transferred, implantation rate, pregnancy rate and miscarriage rate.

A summary of perinatal outcome associated with these treatment cycles is presented in Table 2. At this time, 71 deliveries have occurred with no reported abnormalities (81 babies: 48 boys and 33 girls). There were 47 infants from 41 deliveries with day-5 blastocyst, 26 infants from 23 deliveries with day-6 blastocyst, and 8 infants from 7 deliveries with day-7 blastocyst. There were no differences among the three groups in a ratio of male to female, mean gestational age, preterm delivery rate and mean birth weight.

During the period of this study, there were 10 cycles of vitrified-warmed blastocyst transfer those were mix of different days vitrified. In all 10 cycles, two blastocysts were transferred, one on day 5 and one on day 6 vitrified. A total of 20 (10 day-5 and 10 day-6) blastocysts were vitrified and warmed. All 20 blastocysts (100%) survived after warming and were transferred to 10 patients. The pregnancy rate was 50% (5/10) and the implantation rate was 30% (6/20). Of five pregnancies, one pregnancy revealed two sacs and resulted in delivery of normal, healthy male and female infants weighing 2,848 and 3,102 g at birth, at 35 weeks’ gestation. Another four pregnancies were diagnosed as singletons. Of these, one pregnancy spontaneously aborted at 8 weeks of gestation, and the other three pregnancies are ongoing.

## Discussion

We previously reported 6 cases of successful pregnancy (1 delivery, 2 abortions and 3 ongoing) following 11 transfers of vitrified day-7 blastocyst [8]. The current series is an extension of our earlier research and documents 10 cases of successful pregnancy (7 deliveries, 3 abortions) and perinatal outcomes following 19 transfers of vitrified day-7 blastocyst. As far as is known, this is the first published assessment of perinatal outcomes for IVF patients having transfers of vitrified day-5, day-6 and day-7 blastocyst.

Richter *et al*. [9] observed a significantly lower pregnancy rate with cryopreserved day-7 blastocyst transfers compared with that obtained in cryopreserved day-5 and day-6, but this difference was due, at least in part, to the transfer of significantly fewer transferred day-7 blastocysts, and was not statistically significant after accounting for number of embryos per transfer. In the present report, there were no differences among the vitrified day-5, day-6 and day-7 blastocyst transfer groups in the mean number of embryos transferred, pregnancy rate, implantation rate and miscarriage rate. We also compared the perinatal outcome of vitrified day-5, day-6 and day-7 blastocyst transfer groups. There were no differences among the three groups in the mean gestational age, preterm delivery rate and mean birth weight. These results indicated that blastocysts have similar inherent viability regardless of whether they develop by on day 5, 6 or 7.

The pregnancy rate of vitrified day-7 blastocyst transfers of the present report (52.6%, 10/19) was significantly higher than that of fresh day-7 blastocyst transfers of the reports of Gorrill *et al*. (2001) [13] (4%, 1/25) and Utsunomiya *et al*. [14] (10%, 3/31). In addition, Murata *et al.* [15] reported significantly higher clinical pregnancy results were obtained in cryopreserved day-6 blastocyst transfers (55%, 22/40) compared with fresh day-6 blastocyst replacements (11%, 4/37). Asynchrony with endometrial receptively may therefore be responsible for poorer success rates associated with the fresh transfers of slower developing embryos. Embryo cryopreservation, enabling more appropriate synchronization between embryonic and endometrial development, may therefore be an effective alternative to fresh embryo transfer or cycle cancellation when blastocyst expansion is delayed until day 6 or 7 after oocyte retrieval.

In conclusion, the results of the present study suggest that vitrified human day-7 blastocyst transfer can contribute to increasing cumulative pregnancy rates in assisted reproduction. However, it will require more data on vitrified day-7 blastocyst transfer to determine the risk of spontaneous abortions and birth defects from these more slower developing embryos. Until these data are collected and analyzed, vitrified day-7 blastocyst transfer should be approached with caution.

## Figures and Tables

**Table 1.** Cycle characteristics and clinical outcomes as a function of day (after oocyte retrieval) on which blastocysts were vitrified

Day of vitrification	5	6	7
No. of cycles for warming	144	100	19
Mean age of women at vitrification (years) ± SD	33.3 ± 4.7	33.7 ± 4.4	33.7 ± 3.7
Mean age of women at warming (years) ± SD	34.2 ± 4.5	34.5 ± 4.3	35.0 ± 3.7
No. of blastocysts warmed	214	145	28
No. of blastocysts survived (%)	213 (99.5)	142 (97.9)	28 (100.0)
Mean no. of blastocysts transferred ± SD	1.5 ± 0.5	1.4 ± 0.5	1.4 ± 0.5
No. of embryos implanted (%)	90 (42.3)	59 (41.5)	12 (42.9)
No. of clinical pregnancies (%)	77 (53.5)	53 (53.0)	10 (52.6)
No. of miscarriages (%)	18 (23.4)	16 (30.2)	3 (30.0)
No. of ongoing pregnancies	18	14	0
No. of deliveries	41	23	7

**Table 2.** Characteristics of infants conceived after vitrified-warmed blastocyst transfer according to the day (after oocyte retrieval) on which blastocysts were vitrified

Day of vitrification	5	6	7
No. of live born	47	26	8
No. of male (%)	26 (55.3)	16 (61.5)	6 (75.0)
No. of female (%)	21 (44.7)	10 (38.5)	2 (25.0)
Mean gestational age (wk) ± SD	38.4 ± 2.0	38.6 ± 2.0	39.4 ± 2.0
Preterm (<37 wk) (%)	7 (17.1)	4 (17.4)	1 (14.2)
Mean birth weight (g) ± SD	2944 ± 535	2897 ± 426	3088 ± 270
Twins	6	3	1
